# The predictive value of PD‐L1 expression in patients with advanced hepatocellular carcinoma treated with PD‐1/PD‐L1 inhibitors: A systematic review and meta‐analysis

**DOI:** 10.1002/cam4.5676

**Published:** 2023-03-25

**Authors:** Yao Yang, Dongbo Chen, Bigeng Zhao, Liying Ren, Rui Huang, Bo Feng, Hongsong Chen

**Affiliations:** ^1^ Peking University People's Hospital, Peking University Hepatology Institute Beijing Key Laboratory of Hepatitis C and Immunotherapy for Liver Disease Beijing China; ^2^ Laboratory of Hepatobiliary and Pancreatic Surgery Affiliated Hospital of Guilin Medical University Guilin Guangxi China

**Keywords:** biomarker, hepatocellular carcinoma, immune checkpoint inhibitors, meta‐analysis, programmed death‐ligand 1, treatment response

## Abstract

**Background and Aim:**

Programmed death 1 (PD‐1) and programmed death ligand 1 (PD‐L1) inhibitors have transformed the treatment landscape of advanced hepatocellular carcinoma (HCC), but consistent responses are not observed in all patients, and prognostic biomarkers to guide treatment decisions are lacking. We aimed to evaluate the predictive value of PD‐L1 expression in advanced HCC patients treated with PD‐1/PD‐L1 inhibitors.

**Methods:**

A comprehensive search of PubMed, Embase, Web of Science, and the Cochrane Library was conducted. Studies comparing the objective response rate (ORR) and/or disease control rate (DCR) based on the tumor PD‐L1 status of HCC were included.

**Results:**

Eleven studies with 1,330 HCC patients treated with PD‐1/PD‐L1 inhibitors were included. Pooled odds ratio (OR) analysis demonstrated a significantly improved ORR in PD‐L1‐positive patients compared with PD‐L1‐negative patients (OR, 1.86, 95% CI, 1.35–2.55). Similar results were observed in the anti‐PD‐1 treatment (*p* < 0.001) and anti‐PD‐1/PD‐L1 monotherapy (*p* < 0.001) subgroups. The pooled ORRs in the PD‐L1‐positive and PD‐L1‐negative groups were 26% (95% CI, 20%–32%) and 18% (95% CI, 13%–22%), respectively. For DCR, the pooled OR analysis showed no significant difference between PD‐L1‐positive patients and PD‐L1‐negative patients (66% [95% CI, 55%–76%] vs. 69% [95% CI, 62%–76%]; OR, 0.92, 95% CI, 0.59–1.44). The results were consistent across the drug target and combination treatment subgroups.

**Conclusion:**

Positive PD‐L1 expression is associated with a better ORR in advanced HCC patients treated with anti‐PD‐1/PD‐L1‐based therapies. This feature can help to identify HCC patients who will benefit most from PD‐1/PD‐L1 inhibitors.

## Graphical abstract

Not all patients with advanced HCC can benefit from anti‐PD‐1/PD‐L1 therapy, biomarkers for selecting patients for treatment are urgently needed. Tumor PD‐L1 expression was significantly associated with improved ORR in HCC patients. The ORR and DCR in patients with positive PD‐L1 expression were 26% and 66%, versus 18% and 69% in patients with negative PD‐L1 expression.

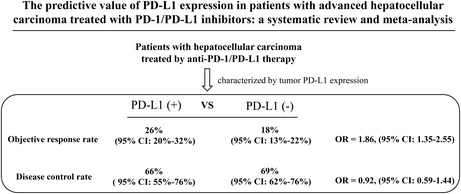

## 1 INTRODUCTION

Hepatocellular carcinoma (HCC) is the most common type of primary liver cancer and is associated with increasing incidence and high mortality.^1^ Due to the occult onset, high degree of malignancy and fast progression of HCC, it is often diagnosed at an intermediate–advanced stage, at which point there is no chance for radical treatment.^2^ Although many systemic therapeutic strategies, such as chemotherapy, radiotherapy, and molecular targeted therapies, have been developed and extensively used in clinics in recent decades, the prognosis of patients with advanced HCC remains unsatisfactory. The recent advent of immunotherapy, especially immune checkpoint inhibitors (ICIs), has dramatically transformed the treatment landscape for advanced HCC.^3^

ICIs are monoclonal antibodies that target tumor immune escape factors, that is, immune checkpoints. Programmed cell death 1 (PD‐1), which is mainly expressed on immune cells, and its ligand programmed death ligand 1 (PD‐L1), which is expressed on tumor cells, are two key immune checkpoints. Using antibodies to block PD‐L1 or PD‐1 can reverse T‐cell dysfunctionality and mount an effective antitumour response.^4^ In recent years, many clinical trials with ICIs for HCC have been conducted, and promising effects have been reported. In 2017 and 2018, the Food and Drug Administration (FDA, USA) granted accelerated approvals for the PD‐1 inhibitors nivolumab and pembrolizumab in advanced HCC based on results from the CheckMate040^5^ and Keynote224^6^ trials, respectively. Recently, camrelizumab and sintilimab have been approved for clinical use for HCC in China.^7^ In addition, the PD‐L1 inhibitor atezolizumab combined with the VEGF inhibitor bevacizumab has been recommended as a first‐line regimen for the treatment of advanced HCC based on the results of the IMbrave150 study.^8^ Other PD‐L1 inhibitors, including avelumab^9^, ^10^ and durvalumab,^11^ are being studied either alone or in combination in HCC.

Although PD‐1/PD‐L1 inhibitors have exhibited dramatic success in cancer treatment, patients show a highly heterogeneous response to the treatment, ranging from sustained complete remission to rapid disease progression. Additionally, such treatments are very expensive and may be associated with a broad spectrum of immune‐related adverse effects.^12^ Therefore, exploring a potential predictive biomarker to identify patients who would benefit the most from ICIs is crucial. Considering the underlying mechanism of PD‐1/PD‐L1 inhibitor treatment, PD‐L1 expression may be the most promising biomarker of the response to ICIs.

Many studies have shown the predictive value of tumor PD‐L1 expression in various cancers, including melanoma,^13^ renal cell cancer,^14^ and non‐small cell lung cancer (NSCLC).^15^, ^16^ However, the value of PDL1 expression in predicting the efficacy of PD1/PDL1 inhibitor monotherapy or combination therapies in HCC remains controversial.^17^, ^18^ The results from CheckMate040 reported that clinical treatment responses were observed irrespective of PD‐L1 expression, but patients with PD‐L1 ≥ 1% tended to exhibit a higher overall response rate (ORR) than patients with PD‐L1 < 1%.^5^ A similar trend was observed in patients with HCC treated with pembrolizumab.^6^ However, in a small cohort of patients with HCC treated with avelumab, no significant difference in ORR was observed between PD‐L1 ≥ 1% and PD‐L1 < 1% patients.^10^ Therefore, we performed this study to assess the value of PD‐L1 expression for predicting the clinical response or survival outcome of HCC patients treated with PD‐1/PD‐L1 inhibitors.

## 2 METHODS

### 2.1 Study design and search strategy

This systematic review and meta‐analysis was conducted in accordance with the Preferred Reporting Items for Systematic Reviews and Meta‐Analyses (PRISMA) guidelines. The protocol was registered on PROSPERO, the international prospective register of systematic reviews (CRD42021288493). We performed a comprehensive literature search of PubMed, EMBASE, Web of Science, and the Cochrane Library from inception through February 28, 2022. The main key search words were “hepatocellular carcinoma”, “PD‐1”, “PD‐L1”, and “clinical trial”. Table S1 shows the detailed literature search strategy. In addition, the references of relevant articles were also manually searched.

### 2.2 Study eligibility

Two reviewers (YY and ZBG) independently screened articles for inclusion, and disagreements were resolved by discussion and consensus and subsequently confirmed by a third reviewer (CDB). The inclusion criteria were as follows: (1) Studies of patients with advanced HCC treated with PD‐1 or PD‐L1 inhibitors were included. (2) The PD‐L1 status of the included patients was examined by immunohistochemistry staining methods. The threshold for PD‐L1 positive or negative expression for HCC was defined as PD‐L1‐stained cells accounting for 1% of tumor cells, tumor‐infiltrating immune cells, or both. Studies with other cut‐off values were only included in the qualitative analysis. (3) Studies reporting at least one objective evaluation of the following outcomes were included: ORR, disease control rate (DCR), progression‐free survival (PFS), or overall survival (OS) according to different PD‐L1 statuses. (4) Clinical trials irrespective of trial phase were included. The exclusion criteria were as follows: (1) studies of patients with multiple malignancies or metastatic liver tumors in addition to HCC; (2) observational studies, reviews, meta‐analyses, comments, case reports, editorials and letters; and (3) studies not published in English. When multiple studies reported data from the same clinical trial, the higher quality, or most complete reporting study, or both studies were included.

### 2.3 Data extraction and quality assessment

Data extraction and quality assessment of the included studies were carried out by the same two authors (YY and ZBG). Any disagreements were resolved by discussion and consensus. The following information was extracted: first author name; trial name, registration number, and phase; line of treatment; study region; PD‐1 or PD‐L1 inhibitor(s) used; drug dose; method for PD‐L1 detection; number of patients; characteristics of the patients and tumors; median follow‐up time; ORR; DCR; OS; PFS, etc. We attempted to contact the authors through email when data were insufficient or missing.

The risk of bias for each study was assessed by the Cochrane Collaboration Tool.^19^ The assessment items included selection bias, performance bias, attrition bias, detection bias, reporting bias, and other biases.

### 2.4 Statistical analysis

The meta‐analysis was performed with STATA 16.0 according to the Cochrane Handbook for Systematic Reviews of Interventions.^20^ We extracted data from the anti‐PD‐1/PD‐L1 treatment arm to calculate response rates (ORR and DCR). ORR was defined as the portion of patients who achieved complete response and partial response, while DCR was defined as the ORR plus the stable disease rate. The odds ratio (OR) and 95% confidence interval (CI) were calculated to compare the ORR and DCR between different PD‐L1 expression groups. If a trial evaluated the same PD‐1/PD‐L1 inhibitors with different doses or with different combination therapies, we considered each to be a single study cohort and analyzed it accordingly.

We used the Cochran's Q test and Higgins' I^2^ statistic to determine the heterogeneity between studies. Fixed or random effects models were selected to pool data according to the level of heterogeneity. The fixed effects model was used when *p* > 0.05 and *I* ^2^ < 50%. Otherwise, the random effects model was selected. A Z‐test was used to determine the significance of pooled ORs, and *p* < 0.05 was considered statistically significant.

Subgroup analysis was conducted by drug target, therapy type (combination therapy or monotherapy), drug and line of treatment. Sensitivity analysis was performed to observe the impact of each study or a certain drug on the overall results. Funnel plots and Egger's and Begg's tests were performed to assess publication bias.

## 3 RESULTS

### 3.1 Study selection and characteristics

Our search strategy identified a total of 5,625 studies. After removing duplicates, 4124 articles were screened. Thirteen studies that evaluated the effects of anti‐PD‐1/PD‐L1 therapy for HCC on clinical responses or survival by PD‐L1 status were ultimately included in the qualitative analysis.^5^, ^6^, ^7^, ^9^, ^10^, ^11^, ^21^, ^22^, ^23^, ^24^, ^25^, ^26^, ^27^ The study selection process is presented in Figure S1. Since the JVDJ trial^26^ defined high or low PD‐L1 expression with a cut‐off value of 25% tumor or immune cell staining for the HCC cohort, it was only included in the qualitative analysis. El‐Khoueiry et al.^5^ and Sangro et al.^27^ reported outcomes of the same patient cohorts from Checkmate040, but Sangro et al reported the OS outcome by PD‐L1 status for patients, so it was included in the qualitative analysis. Ultimately, 11 studies with 1330 patients with advanced HCC were included in the meta‐analysis. All tumors were advanced, metastatic, or unresectable. Of the included patients, 459 (34.5%) patients were PD‐L1 positive, and 871 (65.5%) patients were PD‐L1 negative; 845 (63.5%) patients were treated with an ICI alone, and 485 (36.5%) patients were treated with ICI‐based combinations. Table 1 demonstrates the main characteristics of the included trials, and the characteristics of the included patients are provided in Table S2. All included trials were judged to have a high risk of performance bias for blinding of participants and staff because they were open‐label trials. The details of the quality assessment are presented in Table S3.

**TABLE 1:** Characteristics of the included trials.

Study (year)	Trial name	Registration number	Trial Phase	Region/Center	Line of therapy	Drug name (drug target)	Doses	Combination therapy	PD‐L1 antibody clone	IHC scoring method	PD‐L1 cutoff	Sample	Median follow‐up time	Response evaluation
2017, El‐Khoueiry	CheckMate 040	NCT01658878	1/2	Globally, multicenter	1st or 2nd	Nivolumab (PD‐1)	0.1‐10 mg/kg Q2W; 3 mg/kg Q2W	NA	clone 28–8	Type I	1%	44 in dose escalation cohort, 174 in dose expansion cohort	NA	RECIST v1.1
2018, Zhu	KEYNOTE‐224	NCT02702414	2	Globally, multicenter	2nd	Pembrolizumab (PD‐1)	200 mg Q3W	NA	22C3	Type I	1%	52	12.3 (IQR, 7.6–15.1)	RECIST v1.1
2019, Feun	NA	NCT02658019	2	USA, single center	2nd	Pembrolizumab (PD‐1)	200 mg Q3W	NA	22C3	NA	1%	10	17 (95% CI, 8.0–22.0)	RECIST v1.1
2020, Lee, M.S	GO30140	NCT02715531	1b	Globally, multicenter	1st	Atezolizumab (PD‐L1)	1200 mg Q3W	Bevacizumab	SP263	Type III	1%	86 in group A 95 in group F	12.4 (IQR, 8.0–16.2)	RECIST v1.1
2020, Qin	NA	NCT02989922	2	China, multicenter	2nd or later	Camrelizumab (PD‐1)	3 mg/kg Q2‐3 W	NA	SP142	Type I	1%	30	NA	RECIST v1.1
2020, Yau	CheckMate 040	NCT01658878	1/2	Globally, multicenter	2nd	Nivolumab (PD‐1)	1 mg/kg Q3W, 3 mg/kg Q3W, 3 mg/kg Q2W	Ipilimumab	clone 28–8	Type I	1%	49 in arm A 48 in arm B 48 in arm C	NA	RECIST v1.1
2021, Kelley	NA	NCT02519348	1/2	Globally, multicenter	2nd	Durvalumab (PD‐L1)	1500 mg D4W	Tremelimumab	SP263	Type III	1%	65 in T300 + D 90 in D 72 in T75+D	NA	RECIST v1.1
2021, Kudo	VEGF Liver 100	NCT03289533	1b	Japan, multicenter	2nd or later	Avelumab (PD‐L1)	10 mg/kg Q2W	Axitinib	SP263	Type II	1%	20	Minimum 18.0	RECIST v1.1
2021, Lee, D.W	NA	NCT03389126	2	Korea, single center	2nd	Avelumab (PD‐L1)	10 mg/kg Q2W	NA	SP263	Type I	1%	27	13.9	RECIST v1.1
2021, Yau	CheckMate 459	NCT02576509	3	Globally, multicenter	1st	Nivolumab (PD‐1)	240 mg Q2W	NA	NA	Type I	1%	366	Minimum 22.8	RECIST v1.1
2021, Xu	RESCUE	NCT03463876	2	China, multicenter	1st or 2nd	Camrelizumab (PD‐1)	200 mg or 3 mg/kg Q2W	Apatinib	22C3	Type I	1%	54	16.7 for first‐line; 14.0 for second‐line	RECIST v1.1

Abbreviations: IHC, immunohistochemistry; NA, not available; Type I, PD‐L1 expression on tumor cells; Type II, PD‐L1 expression on tumor‐infiltrating immune cells; Type III, PD‐L1 expression on tumor cells and/or tumor‐infiltrating immune cells.

### 3.2 Meta‐analysis outcomes

#### 3.2.1 Objective response rate

**FIGURE 1:**
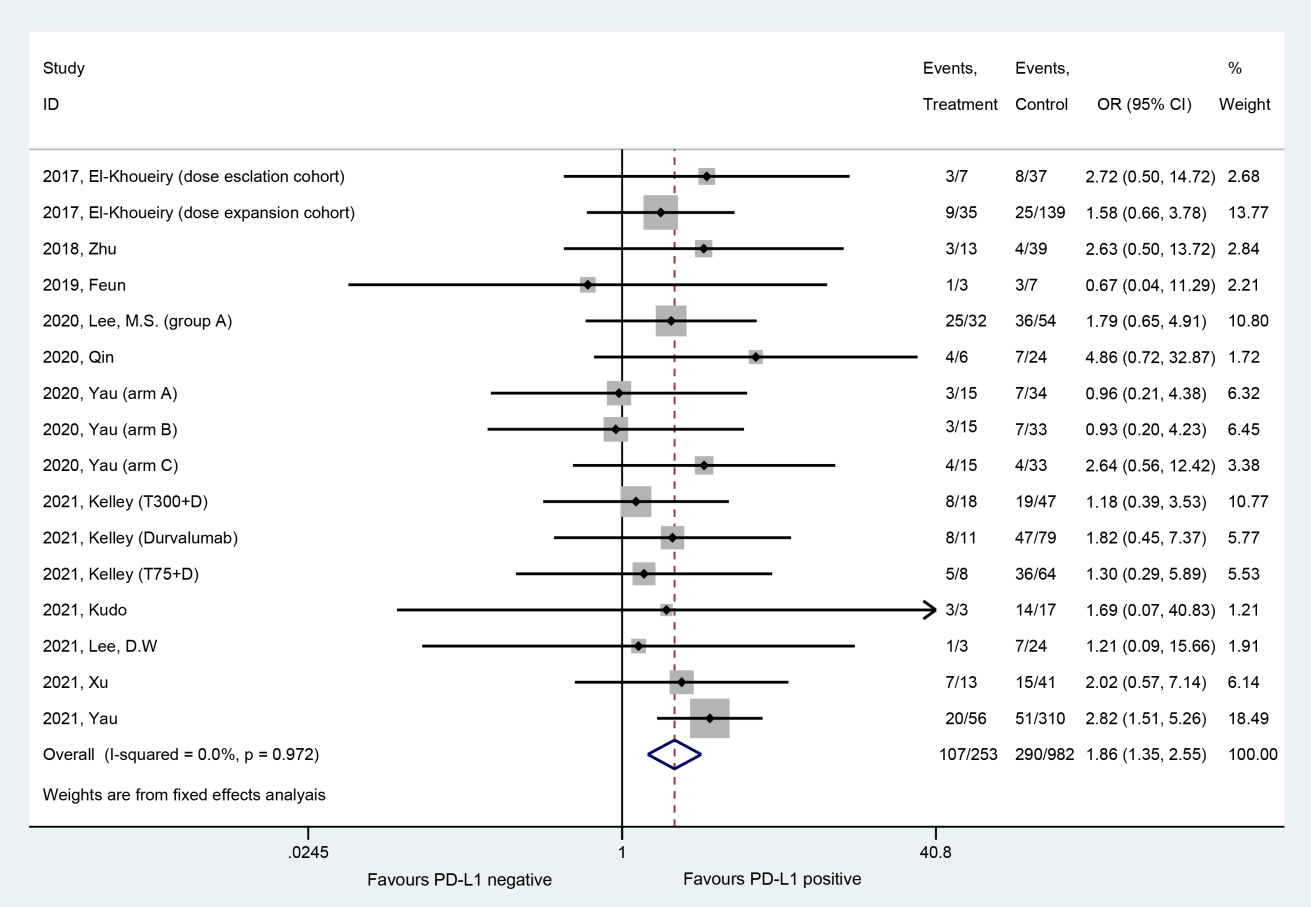
Forest plot of the comparison of ORR between the PD‐L1‐positive and PD‐L1‐negative groups.

Eleven studies with 16 patient cohorts reported the ORR according to PD‐L1 expression among patients with HCC.^5^, ^6^, ^7^, ^9^, ^10^, ^11^, ^21^, ^22^, ^23^, ^24^, ^25^ A total of 1,235 patients (397 patients were PD‐L1 positive, 838 patients were PD‐L1 negative) were included in the meta‐analysis. The pooled OR analysis revealed a significantly improved ORR in patients with PD‐L1‐positive HCC compared with patients with PD‐L1‐negative HCC (OR, 1.86, 95% CI, 1.35–2.55; *p* < 0.001) (Figure 1). The heterogeneity between studies was extremely low (Cochrane's Q test *p* = 0.972, *I* ^2^ = 0.0%), so the fixed effects model was used. The pooled ORRs in the PD‐L1‐positive group and PD‐L1‐negative group were 26% (95% CI, 20%–32%) and 18% (95% CI, 13%–22%), respectively.

**FIGURE 2:**
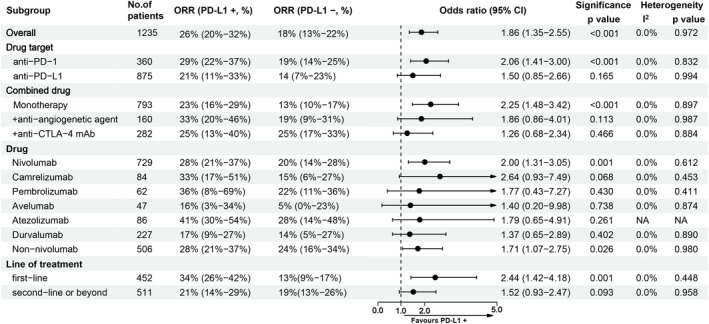
Subgroup analyses for the comparison of ORR between patients with positive PD‐L1 expression and negative PD‐L1 expression.

Figure 2 summarizes the results of the subgroup analyses for ORR. For all subgroups, including drug target (PD‐1 or PD‐L1), therapy type (monotherapy or combination therapy), drug type and different lines of treatment subgroups, the PD‐L1‐positive HCC patients had a higher ORR than the PD‐L1‐negative HCC patients; statistical significances were observed in the PD‐1 target subgroup, monotherapy subgroup, nivolumab subgroup, and first‐line treatment subgroup.

#### 3.2.2 Disease control rate

**FIGURE 3:**
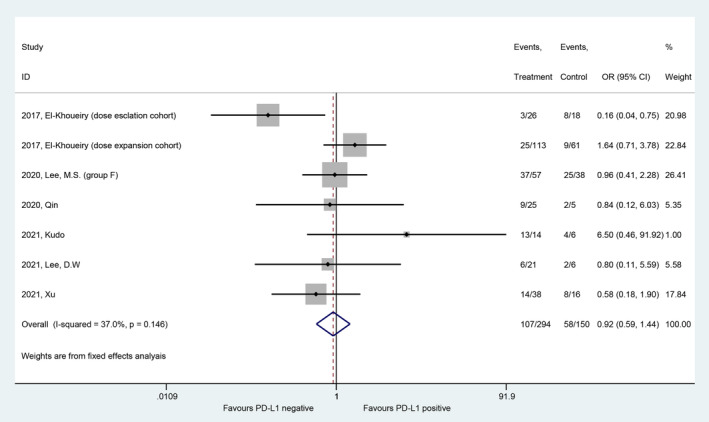
Forest plot of the comparison of DCR between the PD‐L1‐positive and PD‐L1‐negative groups.

**FIGURE 4:**
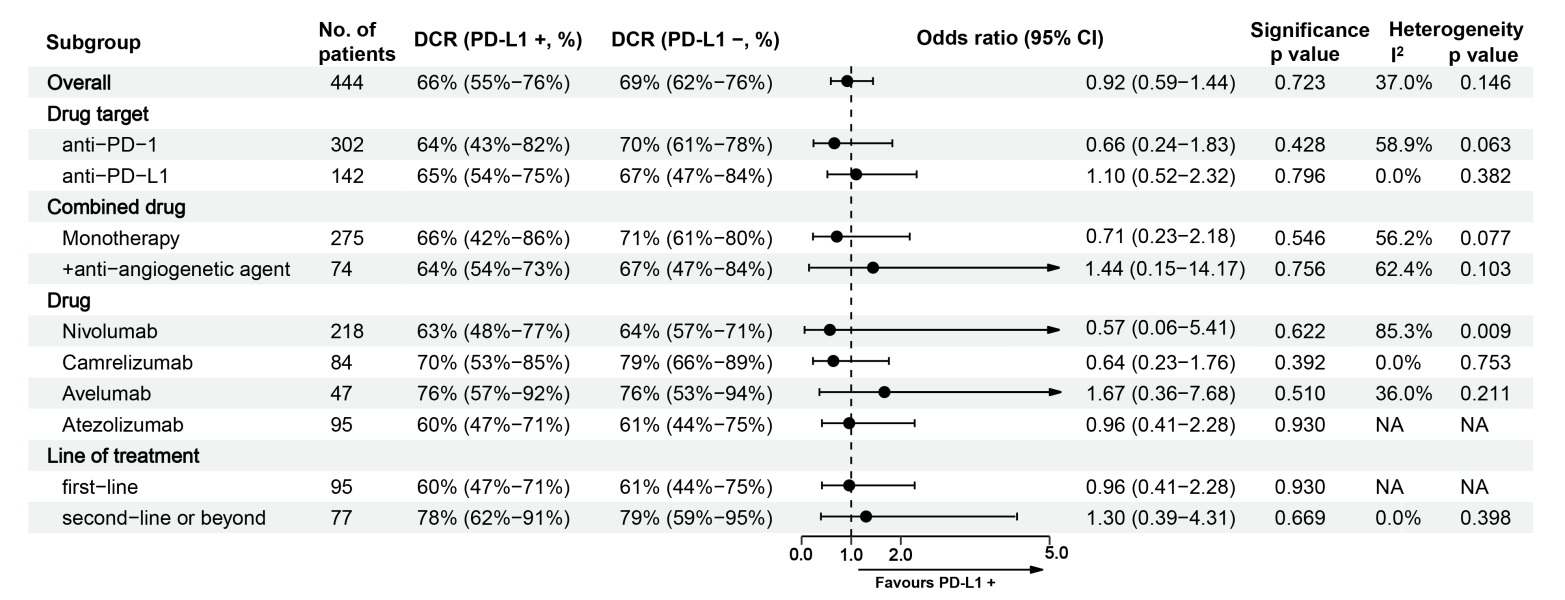
Subgroup analyses for the comparison of DCR between patients with positive PD‐L1 expression and negative PD‐L1 expression.

Six studies with seven patient cohorts reported outcomes of disease control according to PD‐L1 expression.^5^, ^7^, ^9^, ^10^, ^23^, ^25^ A total of 444 patients (165 patients were PD‐L1 positive, 279 patients were PD‐L1 negative) were included in the meta‐analysis. The pooled OR analysis demonstrated no significant difference in DCR between PD‐L1‐positive patients and PD‐L1‐negative patients (OR, 0.92; 95% CI, 0.59–1.44, *I* ^2^ = 0.0%) (Figure 3). This finding was consistent in all subgroups. Figure 4 shows the detailed results of subgroup analyses for DCR. The pooled DCRs in the PD‐L1‐positive group and PD‐L1‐negative group were 66% (95% CI, 55%–76%) and 69% (95% CI, 62%–76%), respectively.

#### 3.2.3 Sensitivity analyses and publication bias

First, sensitivity analysis was performed for ORR and DCR by deleting each study individually, and the results did not change significantly. In addition, to rule out the effect of nivolumab on the overall ORR, sensitivity analysis was also performed by excluding patients treated with nivolumab, and the ORR did not change significantly.

The funnel plots suggested no evidence of publication bias for ORR and DCR, as shown in S2 and S3, and the results of Begg's test (*p* = 0.964) and Egger's test (*p* = 0.208) also showed no publication bias.

#### 3.2.4 Overall survival and progression‐free survival

Six studies reported OS and/or PFS outcomes based on PD‐L1 status. However, due to the relatively small number of eligible studies and the lack of essential data for meta‐analysis, only quantitative analysis was performed for OS and PFS. The median OS time in the PD‐L1‐positive group ranged from 10.2 to 28.1 months, while the median OS time in the PD‐L1‐negative group ranged from 8.0 to 22.2 months. In addition, the results of the Checkmate040 trial showed that tumor PD‐L1 positivity was significantly associated with a longer OS (*p* = 0.032). The median PFS time was similar in the two groups. The detailed results are summarized in Table 2.

**TABLE 2:** Summary of studies that reported overall survival and progression‐free survival by tumor PD‐L1 status.

Study (year)	Trial name	Drug	Median OS time (95% CI, month)		Median PFS time (95% CI, month)	
			PD‐L1 positive	PD‐L1 negative	PD‐L1 positive	PD‐L1 negative
2020, Sangro	Checkmate040	Nivolumab	28.1 (14.2‐NE)	16.6 (14.2–20.2)	NA	NA
2020, Lee.M.S‐group F	GO30140	Atezolizumab + Bevacizumab	NA	NA	5.6 (2.6‐NE)	5.7 (2.2‐NE)
2020, Lee.M.S‐group F	GO30140	Atezolizumab	NA	NA	2.1 (1.7–5.6)	4.0 (1.8–7.4)
2020, Yau‐arm A	Checkmate040	Nivolumab + Iipilimumab	18.8 (2.5‐NE)	22.2 (9.4‐NE)	NA	NA
2020, Yau‐arm B	Checkmate040	Nivolumab + Iipilimumab	10.2 (2.0‐NE)	12.5 (8.0–16.5)	NA	NA
2020, Yau‐arm C	Checkmate040	Nivolumab + Iipilimumab	NE (0.6‐NE)	10.4 (6.8–33.0)	NA	NA
2021, Kudo	VEGF Liver 100	Avelumab + Axitinib	NE (8.7‐NE)	8.0 (6.1‐NE)	5.6 (1.9–9.2)	5.5 (1.8–9.2)
2021, Yau	Checkmate459	Nivolumab	16.1 (8.4–22.3)	16.7 (13.9–18.6)	3.8 (2.1–7.6)	3.6 (2.4–3.8)
2020, Bang	JVDJ	Durvalumab + Ramucirumab	16.5 (5.1–18.4)^a^	5.7 (1.9–14.4)^a^	5.6 (1.5‐NE)^a^	2.8 (0.7–5.5)^a^

Abbreviations: NA, not available; NE, not estimable.

^a^ In Bang's study, HCC patients were divided into high PD‐L1 cohort and low PD‐L1 cohort by cut‐off value of 25%.

## 4 DISCUSSION

We performed a comprehensive systematic review and meta‐analysis to evaluate the value of PD‐L1 expression for predicting the clinical response to PD‐1/PD‐L1 inhibitors in clinical trials including patients with advanced HCC. The meta‐analysis revealed that in HCC patients treated with PD‐1/PD‐L1 inhibitors, a significantly higher ORR was observed in PD‐L1‐positive patients than in PD‐L1‐negative patients (26% vs. 18%). The meta‐analysis indicated that there was no significant difference between the two patient populations in DCR (66% vs. 69%).

Among a variety of potential prognostic biomarkers for the efficacy of anti‐PD‐1/PD‐L1 therapy, PD‐L1 expression on tumor and/or immune cells is one of the most plausible indicators. Currently, immunohistochemically positive or high expression of PD‐L1 is sometimes a prerequisite for the use of anti‐PD‐1/PD‐L1 immunotherapy in several types of malignant tumors. For example, regarding triple‐negative breast cancer and NSCLC, the PD‐L1 inhibitor atezolizumab and the PD‐1 inhibitor pembrolizumab are only indicated for patients whose tumors express PD‐L1.^28^, ^29^ Some meta‐analyses have also shown a correlation between PD‐L1 expression and improved response rates for melanoma,^30^ renal cell carcinoma,^14^ and urothelial carcinoma.^31^ However, no study has explored the value of PD‐L1 expression in predicting the efficacy of PD‐1/PD‐L1 inhibitor therapy. To the best of our knowledge, our study is the first study on this topic to date. Our systematic review and meta‐analysis, including 1,330 patients with HCC from 11 prospective clinical trials, found that PD‐L1 expression is significantly associated with an improved objective response to anti‐PD‐1/PD‐L1 therapy. In addition, heterogeneity between studies was low, and sensitivity analyses suggested that the pooled result was stable. Possibly due to the limited number of analyzed patients, the difference in ORR between PD‐L1‐positive and PD‐L1‐negative patients treated with PD‐L1 inhibitors did not reach statistical significance.

Our subgroup analyses indicated that positive PD‐L1 expression was significantly associated with a higher ORR than negative PD‐L1 expression in patients treated with anti‐PD‐1/PD‐L1 monotherapy. However, in patients treated with anti‐PD‐1/PD‐L1‐based combination therapy, positive PD‐L1 expression was associated with a higher ORR than negative PD‐L1 expression, but the difference did not reach statistical significance. In recent years, many preclinical models and clinical trials have demonstrated that combining anti‐angiogenic agents (including tyrosine kinase inhibitors and anti‐VEGFR monoclonal antibodies) with ICIs can modulate the tumor microenvironment and immunometabolism and increase PD‐L1 expression in tumors, thus leading to a strong and sustained antitumour effect.^32^, ^33^, ^34^ Another promising combination therapy compromises PD‐1/PD‐L1 inhibitors plus inhibitors targeting another immune checkpoint, cytotoxic T lymphocyte‐associated protein 4 (CTLA‐4). The combination of PD‐1/PD‐L1 inhibitors and CTLA‐4 inhibitors (e.g., nivolumab+ipilimumab and durvalumab+tremelimumab) can stimulate antitumour immune responses through different and complementary mechanisms targeting different signaling pathways. These addictive or synergistic effects of antiangiogenic or anti‐CTLA‐4 agents may have resulted in a lack of significant difference in ORR between the PD‐L1‐positive and PD‐L1‐negative groups. Further studies are required to explore the predictive value of PD‐L1 expression in the response to PD‐1/PD‐L1‐based combination therapy.

Our meta‐analysis supports the predictive value of tumor PD‐L1 expression for HCC patients treated with PD‐1/PD‐L1 inhibitors. One noteworthy point is that therapeutic responses were also seen in PD‐L1‐negative patients, and this should be considered when formulating treatment plans. The underestimation of PD‐L1 expression by sample error in heterogeneous tumors, dynamic changes in PD‐L1 expression under different tumor microenvironments, and possible expression of PD‐L2 might explain this result.^35^, ^36^, ^37^, ^38^ Of course, further studies are needed to fully explore the possible mechanism. Therefore, the expression of PD‐L1 in tumor tissues may not serve as an independent marker for eligibility for anti‐PD‐1/PD‐L1 treatment, but it may represent a marker of better therapeutic response.

When comparing DCR between the patients with positive and negative PD‐L1 expression, no significant difference was found, and this result was consistent across subgroups. Since the PD‐L1‐positive patients possessed a higher ORR than the PD‐L1‐negative patients, the lack of difference in DCR between these two patient populations might indicate a better stable disease rate in PD‐L1‐negative patients. However, it is important to note the correlation between tumor PD‐L1 expression and clinical and histological features. Several studies have shown that positive tumor PD‐L1 expression is associated with more aggressive biological behaviors (such as high serum AFP levels, poor differentiation, and vascular invasion).^39^, ^40^ Therefore, patients with negative PD‐L1 expression might be less prone to tumor progression. These findings may explain the lack of difference in DCR, but additional studies are necessary.

Before PD1/PD‐L1 immunotherapy was used in the HCC field, several studies showed that positive PD‐L1 expression is associated with poor DFS and OS in HCC patients.^41^, ^42^ However, a post hoc analysis of Checkmate040 with 195 HCC patients treated with nivolumab showed that patients with PD‐L1 ≥ 1% had significantly better OS than patients with PD‐L1 < 1% (28.1 months vs. 16.6 months, *p* = 0.032). In addition, Bang et al. reported that durvalumab and ramucirumab treated patients with high PD‐L1 expression had a better prognosis than patients with low PD‐L1 expression when a cut‐off value of 25% was used.^26^ These results again demonstrate the clinical benefit that PD‐1/PD‐L1 inhibitors can bring to advanced HCC patients. However, based on the latest released results of Checkmate459, tumor cell PD‐L1 expression was not predictive for OS or PFS. To date, comparisons of the long‐term outcomes of patients treated with ICI therapies based on PD‐L1 expression are still insufficient. Furthermore, although several studies have compared such outcomes, the follow‐up duration was not long enough to observe possible differences. Therefore, a meta‐analysis could not be performed and we comprehensively summarized the correlation of PD‐L1 expression and survival of HCC patients who underwent PD‐1/PD‐L1 inhibitors. Of course, multiple clinical trials assessing the efficacy of ICIs in HCC are ongoing, and we believe that more relevant results will be reported in the near future.

There are several limitations in our study. This meta‐analysis was carried out at the trial level, and individual patient clinicopathological characteristics and response data were not available. Furthermore, most of the trials were at an early phase for evaluating efficacy and safety, and the data presented in these trials may need to be further validated in subsequent large‐sample studies. Finally, the sample size in some subgroups was small. Further high‐quality RCTs with large sample sizes are needed.

## 5 CONCLUSION

This systematic review and meta‐analysis shows that positive PD‐L1 expression is associated with a better ORR in advanced HCC patients treated with PD‐1 or PD‐L1 inhibitors. PD‐L1 expression may help to identify HCC patients who will benefit most from PD‐1/PD‐L1 inhibitors. Further studies are needed to provide more evidence.

## AUTHOR CONTRIBUTIONS

**Yao Yang:** Conceptualization (equal); data curation (lead); formal analysis (equal); investigation (lead); methodology (lead); project administration (equal); software (equal); writing – original draft (lead). **Dongbo Chen:** Conceptualization (equal); funding acquisition (equal); supervision (equal); writing – review and editing (equal). **Bigeng Zhao:** Data curation (equal); methodology (equal); software (equal). **Liying Ren:** Data curation (equal); software (equal). **Rui Huang:** Data curation (equal); investigation (equal); methodology (equal). **Bo Feng:** Conceptualization (equal); project administration (equal); writing – review and editing (equal). **Hongsong Chen:** Conceptualization (equal); project administration (equal); writing – reviewing and editing (equal).

## FUNDING INFORMATION

The work was supported in part by a grant from the National Key Sci‐Tech Special Project of China (grant number: 2018ZX10302207); the Peking University Medicine Seed Fund for Interdisciplinary Research (grant number: BMU2021MX007) and the Fundamental Research Funds for the Central Universities; and the Peking University People's Hospital Scientific Research Development Funds (grant number: RDX2020‐06).

## CONFLICT OF INTEREST STATEMENT

The authors have no conflict of interest to declare.

## Supporting information


Figure S1

Figure S2

Figure S3

Table S1‐S3

## Data Availability

All data generated or analyzed during the meta‐analysis are included in the published article and its supplementary materials.
