# Supplementary figures and images for: The predictive value of PD‐L1 expression in patients with advanced hepatocellular carcinoma treated with PD‐1/PD‐L1 inhibitors: A systematic review and meta‐analysis
Source: Cancer Med. 2023 Mar 25;12(8):9282–92. doi: 10.1002/cam4.5676 (PMC10166972; doi:10.1002/cam4.5676)

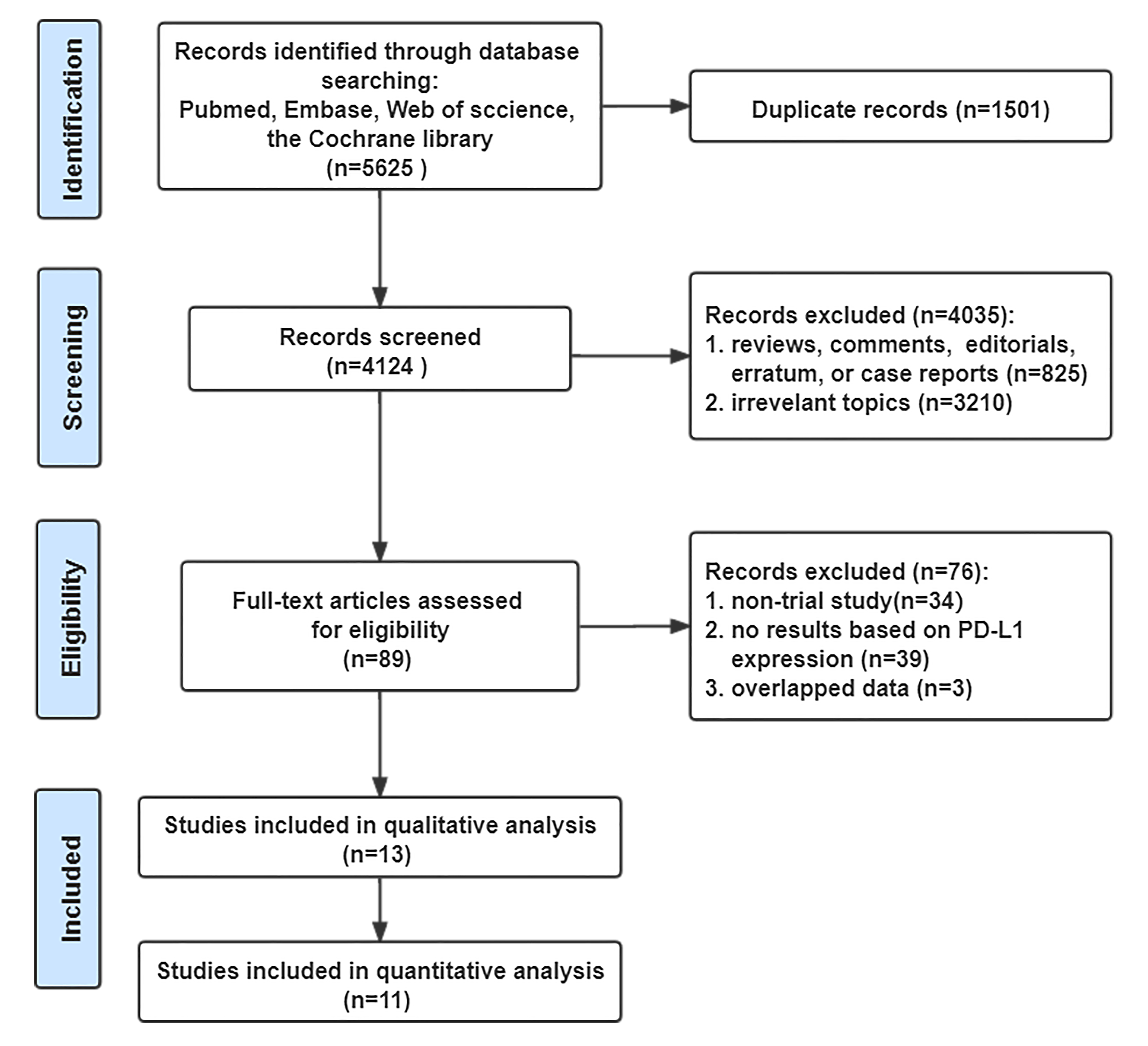

Supplement: Supplementary file 1 — Figure S1 [file CAM4-12-9282-s001.tif]

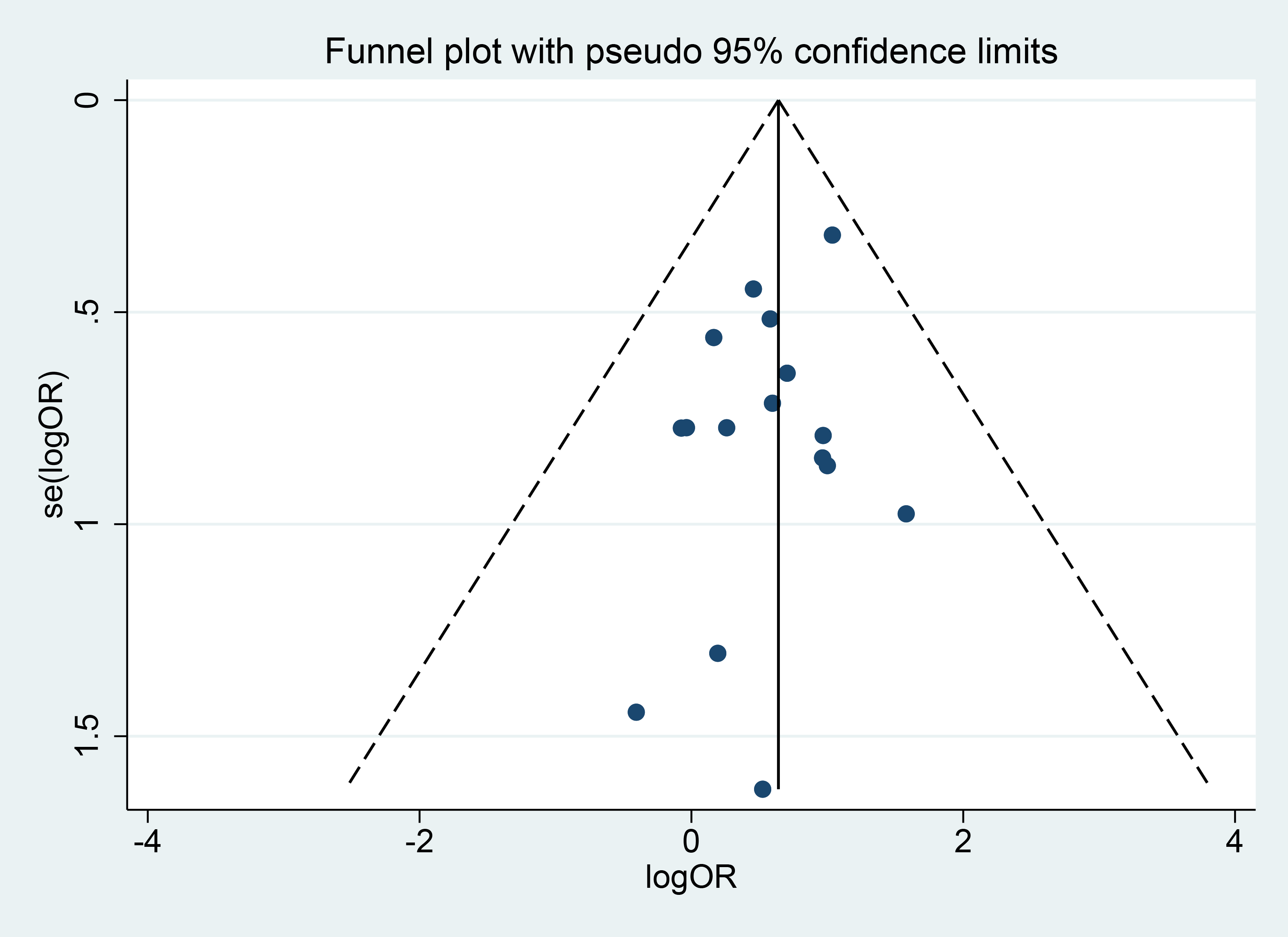

Supplement: Supplementary file 2 — Figure S2 [file CAM4-12-9282-s002.tif]

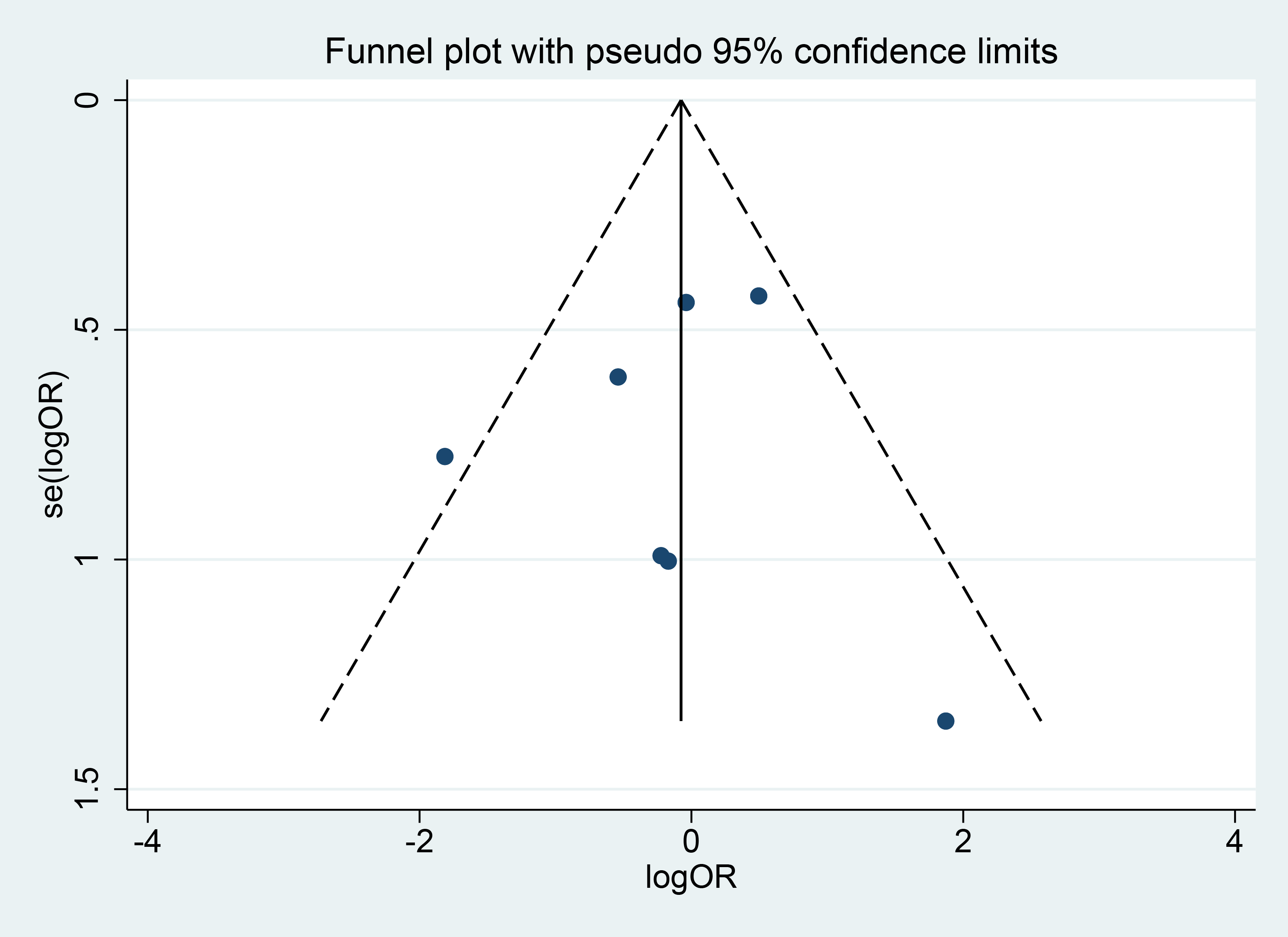

Supplement: Supplementary file 3 — Figure S3 [file CAM4-12-9282-s003.tif]
