# Supplementary material for: The predictive value of PD‐L1 expression in patients with advanced hepatocellular carcinoma treated with PD‐1/PD‐L1 inhibitors: A systematic review and meta‐analysis
Source: Cancer Med. 2023 Mar 25;12(8):9282–92. doi: 10.1002/cam4.5676 (PMC10166972; doi:10.1002/cam4.5676)
Supplement: Supplementary file 4 — Table S1‐S3 [file CAM4-12-9282-s004.docx]

**Table S1. Research strategy.**

**Pubmed**

| No. | Search detail |
| --- | --- |
| #1 | “carcinoma, hepatocellular” [MeSH] |
| #2 | ((((carcinoma*) OR (cancer*)) OR (tumor*)) OR (neoplasm*)) OR (malign*) |
| #3 | (hepatocell*) OR (liver) |
| #4 | #2 and #3 |
| #5 | #1 or #4 |
| #6 | "Immune Checkpoint Inhibitors"[MeSH] |
| #7 | ((Immunocheckpoint inhibitor*[Title/Abstract]) OR (Immune checkpoint inhibitor*[Title/Abstract]))) |
| #8 | (((((((((((((((((((programmed death 1[Title/Abstract]) OR (programmed cell death protein 1[Title/Abstract])) OR (PD-1[Title/Abstract])) OR (PD 1[Title/Abstract])) OR (programmed death-ligand 1[Title/Abstract])) OR (PD-L1[Title/Abstract])) OR (PD L1[Title/Abstract])) OR (anti-PD-1[Title/Abstract])) OR (anti-PD-L1[Title/Abstract])) OR (nivolumab[Title/Abstract])) OR (pembrolizumab[Title/Abstract])) OR (avelumab[Title/Abstract])) OR (atezolizumab[Title/Abstract])) OR (camrelizumab[Title/Abstract])) OR (cemiplimab[Title/Abstract])) OR (durvalumab[Title/Abstract])) OR (toripalimab[Title/Abstract])) OR (tislelizumab[Title/Abstract])) OR (sintilimab[Title/Abstract])) OR (spartalizumab[Title/Abstract]) |
| #9 | #6 OR #7 OR #8 |
| #10 | "Clinical Trials as Topic"[Mesh] |
| #11 | (((((((clinical trial[Title/Abstract]) OR (trial[Title/Abstract])) OR (phase 1[Title/Abstract])) OR (phase I[Title/Abstract])) OR (phase 2[Title/Abstract])) OR (phase II[Title/Abstract])) OR (phase 3[Title/Abstract])) OR (phase III[Title/Abstract]) |
| #12 | #10 OR #11 |
| #13 | #5 AND #9 AND 12 |
| Filters: English | |
| Results: 385 articles were found | |

**Embase**

| No. | Search detail |
| --- | --- |
| #1 | 'liver cell carcinoma'/exp |
| #2 | hepatocell* OR liver |
| #3 | carcinoma* OR tumor* OR neoplasm* OR cancer* OR maligan* |
| #4 | #2 AND #3 |
| #5 | #1 or #4 |
| #6 | 'immune checkpoint inhibitor'/exp |
| #7 | 'immunocheckpoint inhibitor*':ti,ab,kw OR 'immune checkpoint inhibitor*':ti,ab,kw |
| #8 | 'programmed death 1':ti,ab,kw OR 'programmed cell death protein 1':ti,ab,kw OR 'pd-1':ti,ab,kw OR pd1:ti,ab,kw OR 'programmed death-ligand 1':ti,ab,kw OR 'pd-l1':ti,ab,kw OR pd-l1:ti,ab,kw OR 'anti pd 1':ti,ab,kw OR 'anti pd l1':ti,ab,kw OR nivolumab:ti,ab,kw OR pembrolizumab:ti,ab,kw OR avelumab:ti,ab,kw OR atezolizumab:ti,ab,kw OR camrelizumab:ti,ab,kw OR cemiplimab:ti,ab,kw OR durvalumab:ti,ab,kw OR toripalimab:ti,ab,kw OR tislelizumab:ti,ab,kw OR sintilimab:ti,ab,kw OR spartalizumab:ti,ab,kw |
| #9 | #6 OR #7 OR #8 |
| #10 | 'clinical trial'/exp |
| #11 | 'clinical trial':ti,ab,kw OR trial:ti,ab,kw OR 'phase 1':ti,ab,kw OR 'phase I':ti,ab,kw OR 'phase 2':ti,ab,kw OR 'phase II':ti,ab,kw OR 'phase 3':ti,ab,kw OR 'phase III':ti,ab,kw |
| #12 | #10 OR #11 |
| #13 | #5 AND #9 AND #12 |
| #14 #5 AND #12 AND #13 AND [english]/lim | |
| Results: 1771 articles were found | |

**Web of science**

| No. | Search detail |
| --- | --- |
| #1 | TS= (carcinoma, hepatocellular) |
| #2 | TS= (hepatocell* OR liver) |
| #3 | TS= (carcinoma* OR cancer* OR tumor* OR neoplasm* OR malign*) |
| #4 | #2 AND #3 |
| #5 | #1 OR #4 |
| #6 | TS= (Immune Checkpoint Inhibitor* OR Immunocheckpoint inhibitor*) |
| #7 | TS= (programmed death 1 OR programmed cell death protein 1 OR PD-1 OR PD 1 OR programmed death-ligand 1 OR PD-L1 OR PD L1 OR anti-PD-1 OR anti-PD-L1 OR nivolumab OR pembrolizumab OR avelumab OR atezolizumab OR camrelizumab OR cemiplimab OR durvalumab OR toripalimab OR tislelizumab OR sintilimab OR spartalizumab) |
| #8 | (#6) OR #7 |
| #9 | TS=(clinical trial OR trial OR phase 1 OR phase I OR phase 2 OR phase II OR phase 3 OR phase III) |
| #10 | ((#5) AND #8) AND #9 |
| #11 | ((#5) AND #8) AND #9 |
| Filter: English | |
| Results: 1697 articles were found | |

**The Cochrane library**

| No. | Search detail |
| --- | --- |
| #1 | Mesh descriptor: [Carcinoma, Hepatocellular] explored all trees |
| #2 | (hepatocell*) OR (liver) |
| #3 | (carcinoma*) OR (cancer*) OR (tumor*) OR (neoplasm*) OR (malignan*) |
| #4 | #2 and #3 |
| #5 | #1 or #4 |
| #6 | MeSH descriptor: [Immune Checkpoint Inhibitors] explode all trees |
| #7 | (immunocheckpoint inhibitor*):ti,ab,kw OR (immune checkpoint inhibitor*):ti,ab,kw |
| #8 | (programmed death 1):ti,ab,kw OR (programmed cell death protein 1):ti,ab,kw OR (PD-1):ti,ab,kw OR (PD1):ti,ab,kw OR (anti-PD-1):ti,ab,kw OR (programmed death-ligand 1):ti,ab,kw OR (PD-L1):ti,ab,kw OR (PDL1):ti,ab,kw OR (anti-PD-L1):ti,ab,kw OR (nivolumab):ti,ab,kw OR (pembrolizumab):ti,ab,kw OR (avelumab):ti,ab,kw OR (atezolizumab):ti,ab,kw OR (camrelizumab):ti,ab,kw OR (cemiplimab):ti,ab,kw OR (durvalumab):ti,ab,kw OR (toripalimab):ti,ab,kw OR (tislelizumab):ti,ab,kw OR (sintilimab):ti,ab,kw |
| #9 | #6 OR #7 OR #8 |
| #10 | MeSH descriptor: [Clinical Trial] explode all trees |
| #11 | (clinical trial):ti,ab,kw OR (trial):ti,ab,kw OR (phase 1):ti,ab,kw OR (phase I):ti,ab,kw OR (phase 2):ti,ab,kw OR (phase II):ti,ab,kw OR (phase 3):ti,ab,kw OR (phase III):ti,ab,kw |
| #12 | #10 OR #11 |
| #13 | #5 AND 9 AND #12 |
| Results: 1772 articles were found | |

**Table S2. characteristics of patients.**

| Study  (year) | Age | Male (%) | HBV/HCV/Non-viral (%) | CP-A (%) | BCLC-A/B/C  (%) | Extrahepatic metastases (%) | Vascular invasion (%) |
| --- | --- | --- | --- | --- | --- | --- | --- |
| 2017, El-Khoueiry  (dose escalation cohort) | 62 (55-69) | 75 | 31.3/20.8/47.9 | 100.0 | NA | 71.0 | 40.0 |
| 2017, El-Khoueiry  (dose expansion cohort) | 65 (56-70) | 80 | 23.8/23.4/52.8 | 98.0 | NA | 67.0 | 29.0 |
| 2018, Zhu | 66 (60-72) | 83 | 15.0/25.0/NA | 94.0 | 0/21.0/79.0 | 62.0 | 19.0 |
| 2019, Feun | 67 (28-29)^＊^ | 86.2 | 17.2/31.0/NA | 96.6 | NA | 72.4 | 31.0 |
| 2020, Lee.M.S- group A | 62 (23-82) ^＊^ | 81.0 | 49.0/30.0/21.0 | 100.0 | 0.0/10.0/90.0 | 71.0 | 53.0 |
| 2020, Lee.M.S-group F-arm Atezolizumab + Bevacizumab | 60 (22-82) ^＊^ | 90 | 57.0/18.0/25.0 | 100.0 | 0.0/10.0/90.0 | 67.0 | 33.0 |
| 2020, Lee.M.S-group F-arm Atezolizumab | 63 (23-85) ^＊^ | 83 | 54.0/17.0/29.0 | 100.0 | 3.0/7.0/90.0 | 66.0 | 42.0 |
| 2020, Qin | 49 (41-59) | 90 | 83.0/NA/NA | 98.0 | 0.0/5.0/95.0 | 82.0 | 12.0 |
| 2020, Yau-arm A | 61 (54-67) | 86 | 56.0/14.0/26.0 | 100.0 | 1.0/8.0/86.0 | 80.0 | 36.0 |
| 2020, Yau-arm B | 65 (56-67) | 76 | 43.0/29.0/22.0 | 96.0 | 0.0/8.0/92.0 | 82.0 | 27.0 |
| 2020, Yau-arm C | 58 (47-65) | 82 | 53.0/24.0/18.0 | 96.0 | 0.0/6.0/94.0 | 86.0 | 39.0 |
| 2021, Kelley-T300+D | 66.0 (26-86) ^＊^ | 86.7 | 36.0/28.0/36.0 | 98.7 | 1.3/17.3/77.3 | 70.7 | 21.3 |
| 2021, Kelley-D | 64.5 (32-89) | 88.5 | 52.9/33.7/13.5 | 98.1 | 1.0/8.7/76.9 | 60.6 | 28.8 |
| 2021, Kelley-T75+D | 61.5 (28-82) | 83.3 | 48.8/36.9/14.3 | 95.3 | 1.2/20.2/67.9 | 57.1 | 23.8 |
| 2021, Kudo | 68.5 (20-84) ^＊^ | 90.9 | 31.8/9.1/NA | 100.0 | 0.0/40.9/59.1 | 50.0 | 27.3 |
| 2021, Lee, D.W | 62 (36-81) ^＊^ | 73.3 | 86.7/10.0/NA | NA | NA | NA | NA |
| 2021, Yau | 65 (57-71) | 85.0 | 31.0/23.0/82.0 | 100.0 | 4.0/14.0/82.0 | 60.0 | 33.0 |
| 2021, Xu | NA | 88.9 | 88.4/0.5/NA | 100.0 | 0.0/17.9/82.1 | 73.0 | 25.8 |

Age are expressed as median (IQR), ^＊^indicates range. NA: not available.

**Table S3. Risk of bias of the included trials**

| Study | Trial name | Randomization | Allocation  concealment | Blinding of  participants and staff | Blinding of outcome assessment | Incomplete  outcome data | Selective outcome  reporting | Other sources  of bias |
| --- | --- | --- | --- | --- | --- | --- | --- | --- |
| 2017, El-Khoueiry | CheckMate 040 | high | high | high | low | low | low | low |
| 2018, Zhu | KEYNOTE-224 | high | high | high | low | low | low | low |
| 2019, Feun | NA | high | high | high | low | low | low | low |
| 2020, Lee, M.S. | GO30140 | high | high | high | low | low | low | low |
| 2020, Qin | NA | low | low | high | low | low | low | low |
| 2020, Yau | CheckMate040 | low | low | high | low | low | low | low |
| 2021, kelley | NA | high | high | high | low | low | low | low |
| 2021, Kudo | VEGF Liver 100 | high | high | high | low | low | low | low |
| 2021, Lee, D.W | NA | high | high | high | low | low | low | low |
| 2021, Xu | RESCUE | high | high | high | low | low | low | low |
| 2021, Yau | CheckMate 459 | low | low | high | low | low | low | low |
| 2020, Bang | JVDJ | high | high | high | low | low | low | low |
